# Intravenous Administration of Standard Dose Tirofiban after Mechanical Arterial Recanalization is Safe and Relatively Effective in Acute Ischemic Stroke

**DOI:** 10.14336/AD.2018.0922

**Published:** 2019-10-01

**Authors:** Zhe Cheng, Xiaokun Geng, Jie Gao, Mohammed Hussain, Seong-Jin Moon, Huishan Du, Yuchuan Ding

**Affiliations:** ^1^Department of Neurology, Beijing Luhe Hospital, Capital Medical University, China; ^2^China-America Institute of Neuroscience, Beijing Luhe Hospital, Capital Medical University, Beijing, China; ^3^Department of Neurosurgery, Wayne State University School of Medicine, Detroit, MI 48201, USA; ^4^Department of Neurointerventional Surgery, Hartford Hospital, CT 06106, USA

**Keywords:** mechanical thrombectomy, Solitaire stent, GP-IIb/IIIa inhibitor, standard dose, intracerebral hemorrhage, vessel recanalization

## Abstract

To investigate the safety and efficacy of intravenous administration of a standard dose of glycoprotein-IIb/IIIa inhibitor tirofiban after vessel recanalization by mechanical thrombectomy in acute ischemic stroke. A consecutive series of patients (n=112) undergoing endovascular ischemic stroke intervention therapy were enrolled. 81 patients were eligible for intravenous (IV) tirofiban treatment for 24 hours after mechanical thrombectomy. The incidence of symptomatic intracranial hemorrhage (sICH), death, National Institutes of Health Stroke Scale (NIHSS) and modified Rankin scale (mRS) were assessed. In the 81 patients receiving tirofiban, 52 patients (64.2%) were treated with IV rt-PA before mechanical thrombectomy. sICH was found in 2 (2.5%) patients with no fatal ICH. Four patients died during 3 months after stroke onset. Successful recanalization with thrombolysis in cerebral infarction (TICI) score ≥2b was achieved in 75 of 81 patients (92.6%) after mechanical thrombectomy. The average number of passes with Solitaire stent retriever was 1.3. At 3 months, 55 of 81 patients (67.9%) had favorable outcomes (mRS<=2). The intravenous application of a standard dose of tirofiban post-Solitaire stent retriever thrombectomy and intravenous thrombolysis appears to be safe and relatively effective in acute ischemic stroke.

Although intravenous rt-PA is the first-line treatment in acute ischemic stroke (AIS) within 4.5 hours of symptom onset, its use is limited by a narrow therapeutic time window and relatively poor revascularization rates. The limitations become especially apparent in proximal large vessel occlusions (LVO) [1-4]. Compared to intravenous rt-PA, endovascular therapy has shown to have an overall higher degree of recanalization rates in LVO and is associated with better short and long-term clinical outcomes. As documented by various recently published studies in the literature, it is the degree of recanalization? in a timely ?fashion that ?determines successful outcomes [5-8].

The recently published five multicenter prospective randomized trials have demonstrated the utility and benefit of second-generation mechanical thrombectomy (MT) devices (primarily stent retrievers) among patients with AIS due to LVO [9-13]. Since 2015, MT with stent-retrievers has been recommended as the first-line treatment in AIS patients with LVO based on the new American Heart Association/American Stroke Association (AHA/ASA) clinical guidelines [14]. The Solitaire device, a self-expanding stent retriever designed for intracranial LVO, showed high rates of successful revascularization (77% with TICI 2b/3) across these studies with a low rate of sICH (0-7.7%) [15]. However, the issue that is faced by numerous patients undergoing vessel recanalization following endovascular therapy is the susceptibility of acute reocclusion or late stenosis during subacute to chronic stages [16]. An inherent risk of endovascular therapies involving MT? (stent retriever/aspiration), angioplasty and stenting is the associated damage to the inner endothelial cell lining of a blood vessel, which leads to local platelet aggregation and subsequent early reocclusion and resultant thromboembolic complications [17, 18].

Tirofiban is an antiplatelet drug and glycoprotein-IIb/IIIa inhibitor that has been widely used in cardiovascular literature showing good safety and efficacy profile. Within the neurovascular literature, tirofiban has been utilized either as stand alone [19] or as a bridge therapy immediately after full dose IV rt-PA [20, 21]in patients with AIS. Some studies have considered the usage of tirofiban to prevent early reocclusion and mitigate thromboembolic complications as an adjunct to endovascular therapy [17, 22]. This application serves as an adjunct to MT in order to prevent local platelet aggregation [23, 24]. However, few studies to date have reported the safety and efficacy of continuous intravenous administration of tirofiban after endovascular therapy with the goal [17] of maintaining vascular patency. In 2013, Lars Kellert et al reported an elevated risk of fatal intracerebral hemorrhage (ICH) and poor outcomes in patients undergoing intravenous tirofiban after AIS endovascular therapy. According to our anecdotal clinical experience, intravenous tirofiban after MT has been safe and effective. Therefore, we conducted a study to identify the safety and efficacy profile of tirofiban after mechanical thrombectomy in AIS.

## MATERIALS AND METHODS

### Patients

We conducted a prospective observational case series study with consecutive patients (n=112) who underwent endovascular therapy for AIS that were hospitalized in the Department of Neurology, Beijing Luhe Hospital from March 1, 2017 to June 1, 2018.

**Figure 1.**
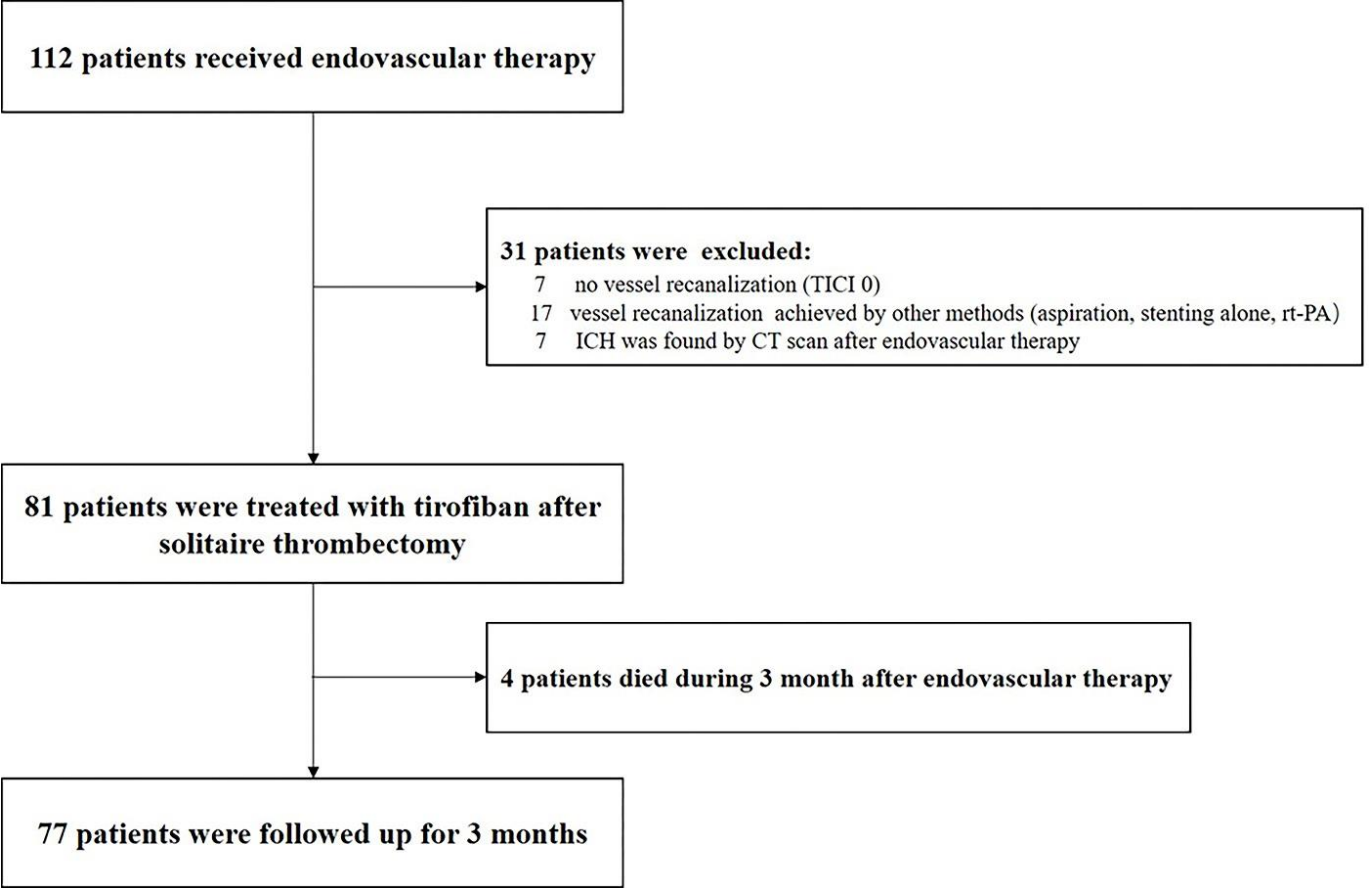
Flowchart of Study.

**Figure 2.**
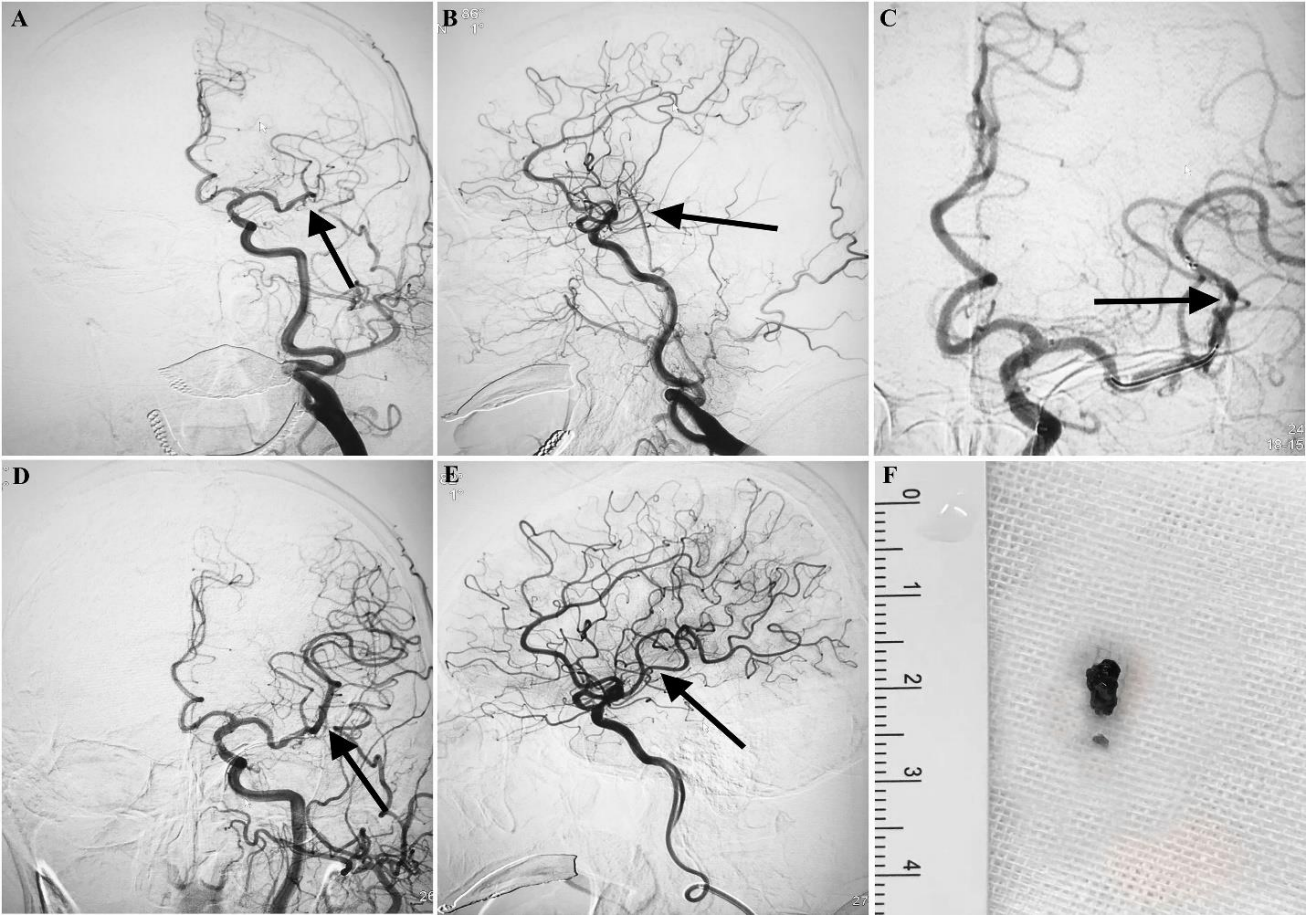
Mechanical thrombectomy procedures. A) DSA reveals persistent L-MCA M2 occlusion segment and fresh thrombus on the occluded segment. B) Lateral view of L-MCA M2 occlusion segment and fresh thrombus on the occluded segment. C) Deployed Solitaire^FR^ retriever (distal and proximal markers: black arrow). D) After one pass with Solitaire^FR^ retriever, successful recanalization was achieved (TICI 3). E: Lateral view of L-MCA after successful vessel recanalization. F) The fresh thrombus removed by Solitaire^FR^ retriever.

The indications for tirofiban administration after endovascular therapy were as follows: 1) establishing a clinical diagnosis of AIS, 2) presence of intracranial LVO, 3) utilization of a second-generation mechanical thrombectomy device (Solitaire stent) for endovascular therapy, 4) vessel recanalization (TICI≥1), 5) no ICH seen on CT head (obtained immediately after MT. Thirty-one patients who were not eligible for intravenous tirofiban treatment were excluded) (Fig. 1).

### Administration of rt-PA and Tirofiban

We routinely initiated rt-PA with a dose of 0.9 mg/kg (10% of the dose was given as a bolus within 1 min followed by a 60 min infusion) while the endovascular intervention was simultaneously being performed. Tirofiban was administered with a bolus of 0.4 μg/kg/min for 30 min (once ICH was ruled out based on post endovascular head CT without contrast), followed by a continuous infusion of 0.1μg/kg/min for 24 hours. Once Tirofiban drip was stopped the patient was transitioned to aspirin (100mg/day) and clopidogrel (75mg/day) daily.

### Mechanical thrombectomy procedures

All procedures were performed under local anesthesia with/without sedative agents. An 8-F sheath was inserted percutaneously under local anesthesia into the right femoral artery. After anticoagulation with IA heparin (80 U/kg), an 8-F guiding catheter together with a 6-F Navien^TM^ intracranial support catheter (ev3, Mansfield, MA, USA) was coaxially introduced into the target vessel. Within the Navien^TM^ catheter, a 0.53mm microcatheter together with a 0.014-inch micro guidewire was navigated distal to the thrombus. This was followed by deployment of the Solitaire stent (SOLITAIRE^TM^ FR, ev3, Mansfield, MA, USA) covering the thrombus. After 10 minutes, the stent and the delivery microcatheter were gently withdrawn through the Navien^TM^ catheter with negative pressure aspiration. To minimize risk of endothelial damage, this procedure was attempted for a maximum of 3 times (Fig. 2).

### Outcome Assessments

A brain computed tomographic (CT) scan without contrast was obtained immediately after endovascular intervention to determine ICH. A second CT head scan or Magnetic Resonance Imaging (MRI) was routinely performed 24 hours after tirofiban administration, or with any sign of neurological deterioration. Vessel re-occlusion was determined based neck CT angiography (CTA) or magnetic resonance angiography (MRA) performed 24 hours after the administration of tirofiban.

ICH was classified into 5 categories according to the European Cooperative Acute Stroke Study II (ECASS 2) [25]: no evidence of hemorrhage, hemorrhagic transformation type I or II (HT I or HT II) and parenchymal hemorrhage type I or II (PH I or PH II). Symptomatic intracerebral hemorrhage (sICH) was defined as PH I or PH II diagnosed in the clinical setting of a ≥4-point increase on the NIHSS score.

The target vessel recanalization was assessed by thrombolysis in cerebral infarct (TICI) scale. Neurological improvement was assessed at day 7, defined as a ≥4-point decrease on the NIHSS after treatment as compared with baseline. The long-term outcome was assessed by mRS scale at 3 months and favorable outcome was defined as mRS score of 0 to 2. Death, cause of death and any other systemic bleeding complications were recorded during 3 months after stroke onset.

### Statistical Analysis

The Kolmogorov-Smirnov test for normality and the equal variance test were performed before any statistical analysis was used. For continuous data, 2-sided *t* test for independent samples was performed to detect differences between groups. For binary data, χ2 or Fisher exact tests were performed when appropriate between-groups. The significance level was set at* P*<0.05. Statistical analysis was performed using SPSS version 19.

**Table 1:** Demographic and clinical characteristics of patient with tirofiban and all patients.

	Tirofiban (n=81)	ALL patients (n=112)
Age, median (range)	64.0 (30,81)	64.6 (30,81)
Sex ratio (male/female)	55/26	71/41
NIHSS at onset, median (range)	18 (9,26)	18 (9,30)
ASPECTS, median (range)	8.9 (6,10)	8.4 (6,10)
Risk factors, n (%)		
Hypertension	64 (79.0)	88 (78.6.)
Diabetes mellitus	18 (22.2)	24 (21.4)
Atrial fibrillation	26 (32.1)	31 (27.7)
Smoking	25 (30.9)	39 (34.8)
Occlusion site, n (%)		
M1	44 (54.3)	55 (49.1)
T-ICA	9 (11.1)	16 (14.3)
P-ICA	9 (11.1)	15 (13.4)
BA	19 (23.5)	26 (23.2)
rt-PA, n (%)	52 (64.2)	71 (63.4)
Stent, n (%)	18 (22.2)	28 (25.0)
NOP, median (min, range)	1.3 (1,4)	1.3 (1,4)
TOR, median (range)	56 (27,125)	57 (27,125)
TTR, median (min, range)	275(60,790)	271 (60,790)
Reocclusion (%)	3 (3.7)	-

NIHSS, the National Institute of Health Scale Score; ASPECT, Alberta Stroke Program Early Computed Tomography Score; mRS, modified Rankin score; M1, middle cerebral artery segment; T-ICA, distal ICA to ACA and MCA; P-ICA, proximal segment of internal carotid artery; BA, basilar artery; IV rt-PA, intravenous recombinant tissue plasminogen activator; NOP, the number of passage of Solitaire stent; TOR, time from onset to recanalization TTR, time from treatment to recanalization.

## RESULTS

Eighty-one patients were treated with tirofiban after target vessel recanalization using Solitaire stent thrombectomy. Baseline demographic and characteristics of the 81 patients who were given tirofiban are summarized in Table 1. The median age was 64.0 years (range 30-81) and NIHSS score at onset was 18 (range 9-26). A total of 62 patients had an occlusion in the anterior circulation, with 19 patients having a posterior circulation occlusion. The average Alberta stroke program early CT (ASPECT) score in patients with the anterior circulation occlusion was 8.9. A total of 52 patients (64.2%) were treated with IV rt-PA before MT. Emergent stenting was performed in 18 patients (22.2%) after MT for treatment of arteriostenosis. In these 18 patients, 9 were extracranial stents in the proximal segment of internal carotid artery; 2 were intracranial stents in middle cerebral artery; 8 were intracranial stents in basilar artery (Severe stenosis was found after recanalization in these 8 patients). Keeping in mind the relatively high mortality rates with basilar artery occlusion (ranging anywhere from 80-100%), the decision was made to perform angioplasty in these 8 patients. The average number of passes (NOP) of Solitaire stent was 1.3. The average time from stroke onset to recanalization (TOR) was 275 minutes. The average device manipulation time from treatment to recanalization (TTR) was 56 minutes. Three patients were found vessel reocclusion at 3 days after MT.

**Table 2:** Clinical outcomes of tirofiban and all patients versus five large randomized control trials.

	TICI ≥2b	1-week NIHSS	ICH	sICH	Fatal ICH	3-month mRS (0-2)	3-month death
Tirofiban (n=81)	92.6%	7	7.4%	2.5%	0	67.9%	4.9%
All patients (n=112)	87.5%	7	8.0%	4.5%	1.8%	61.6%	12.5%
Five RCT	58.7-86%	_	-	0-7.7%	_	32.6-71.4%	9-21%

NIHSS, National Institute of Health Stroke at admission; sICH, symptomatic intracranial hemorrhage; ICH, intracranial hemorrhage; Five RCT, five large randomized control trials

In patients receiving tirofiban after MT (Table 2), successful recanalization (TICI≥2b) was achieved in 75 of 81 patients (92.6%). Six (7.4%) patients with tirofiban were found to have ICH on CT scan at 24 hours after enrollment. Two (2.5%) were classified as sICH and none had fatal ICH. Four (4.9%) patients died during 3 months after stroke onset. NIHSS score at 1 week was 7 (range 0-24). Neurological improvement at 7 days was observed in 64 patients (79.0%) with tirofiban. At 3 months, 55 in 81 patients (67.9%) had favorable outcomes with a mRS of 0 to 2.

**Table 3:** The demographic and clinical characteristics between ICH and no-ICH patients.

	ICH(n=6)	no-ICH(n=75)	P Value
Age (mean ±SD)	63.3±10.8	64.7±10.6	0.871
Hypertension (n, %)	5(83.3%)	59 (79.7%)	1.0 (Continuity correction)
NIHSS at onset (mean ±SD)	18±4.4	17±4.7	0.933
Occlusion (anterior/posterior)	6/0	56/19	0.161 (Fisher)
Stent (n, %)	2 (28.6)	16(21.3)	
IV rt-PA	2 (28.6)	50 (66.7)	0.232 (Continuity correction)
NOP (mean ±SD)	1.2±0.4	1.3±0.6	0.522
TOR (min, mean ±SD)	74±20	55±22	0.048^*^
TTR (min, mean ±SD)	220±50	279±127	0.260

ICH, intracranial hemorrhage; sICH, symptomatic intracranial hemorrhage; NIHSS, the National Institute of Health Scale Score; ASPECT, Alberta Stroke Program Early Computed Tomography Score; IV rt-PA, intravenous recombinant tissue plasminogen activator; NOP, the number of passages of Solitaire stent; TOR, time from onset to recanalization TTR, time from treatment to recanalization. * P< 0.05.

Between patients experiencing no ICH with ICH, no significant difference was found in age, hypertension, NIHSS score at onset, occlusion site (anterior/posterior circulation), stent, IV rt-PA, NOP and TTP. The time from treatment to recanalization in ICH patients was significantly longer than no-ICH patients (P<0.05) (Table 3). Additionally, ASPECT scores in anterior circulation strokes in patients with ICH were significantly lower than no-ICH patients (P<0.05) (Table 4).

In all 112 patients (Table 2), 88.4% achieved successful recanalization (TICI≥2b), which was superior to the mean rate (71%) reported in the five RCT, although an exception was found in SWIFT (88%, same rate as ours) and in EXTEND-IA (86%). Our rate of TICI≥2b was largely superior to other 3 RCT (MR CLEAN FOR 59%, ESCAPE for 72% and REVASCAT for 66%) [26]. In addition, our recanalization rates were also better than those in DAWN (84%) [27]and DEFUSE III (76%) [28]. A total of 61.6% had favorable outcomes with a mRS of 0 to 2, in comparison to the 32.6-71.4% demonstrated in the five large randomized trials. sICH was found in 4.5% of all patients in our study, similar to the 0-7.7% range in the five large randomized trials. A similar rate of low morbidity rate was also observed in our study compared to the five large randomized trials (12.5 versus 9-21%).

**Table 4:** ASPECT Score in anterior circulation stroke between ICH and no-ICH patients.

	ICH (n=6)	no-ICH (n=56)	P Value
ASPECT in anterior circulation stroke(mean ±SD)	7.7±1.8	8.7±1.0	0.045^*^

ASPECT, Alberta Stroke Program Early Computed Tomography Score; ICH, intracranial hemorrhage; sICH, symptomatic intracranial hemorrhage.

* P< 0.05.

## DISCUSSION

Results of our study show a favorable outcome post intracranial arterial thrombectomy of LVO followed by intravenous infusion of tirofiban with low rates of sICH, no fatal ICH and high rate of favorable 3-month clinical outcomes. Thus, intravenous administration of the standard dose of tirofiban post MT and IV rt-PA in AIS with LVO may be seen as a safe and efficacious adjunctive therapy. ?With the new AHA/ASA guidelines mandating use of stent retriever thrombectomy in LVO ischemic strokes, adjunctive therapies such as this to enhance and maintain patency of a recanalized vessel become important.

Although the application of tirofiban has been used in clinical practice for the treatment of patients with coronary disease, there has not been enough clinical evidence in the form of clinical trials to prove the effectiveness of tirofiban in cerebrovascular disease. There had been a study reporting the use of GPIIb/IIIa inhibitors with increased ICH rates in mice [29]. Moreover, a previously conducted review in 2014 reported glycoprotein-IIb/IIIa inhibitors to be associated with a significantly increased risk of sICH [30]. However, conclusions from the existing studies in literature on tirofiban administration in endovascular therapy cannot be made as these have both limited design and small sample sizes [23, 24, 31-33]. Transient IA infusion of an antiplatelet drug after IA thrombolysis or MT have been reported frequently in preventing/minimizing immediate reocclusion and thromboembolic complications [23, 24, 31-33]. Although these studies have shown safety and efficacy of transient IA tirofiban after endovascular therapy, data on its overall efficacy remains relatively insufficient. Enomoto Y et, al reported that reocclusion of recanalized vessels continues to occur up to 24 hours after endovascular therapy [16]. Initial reports ? suggested that continuous intravenous tirofiban ?could decrease reocclusion at 24 hours after intravenous rt-PA (2.4% versus 22.0%) [20]. Thus, by virtue of clinical extension, continuous intravenous tirofiban for a full 24 hours may be more effective to prevent early reocclusion of recanalized vessel after MT. In our study, vessel reocclusion was found in only 3.7% of patients when compared to 7.9% in Enomoto Y’s study [16].

However, the concern of intracranial hemorrhage has been addressed previously. Kellert et al recently reported that intravenous tirofiban was associated with fatal ICH and poor outcomes in AIS treated with MT [17]. In contrast to the non-sICH or fatal ICH in our study, Kellert et al reported 8 sICH cases in 50 patients (16%) and 6 patients (12%) with fatal ICH after tirofiban treatment. On closer analysis, higher degree of poor outcomes and fatal ICH in the Kellert et al study can be attributed to an overall lack of standardized approach in tirofiban administration. Higher rates of hemorrhage can also be due to the underlying endothelial damage from multiple thrombectomy passes [34]. In fact, there was no difference in number of thrombectomy passes between the tirofiban and non-tirofiban group. Furthermore, in the Kellert et al study, a variety of catheters/devices (N=20) were used, all of which again contribute to different degree of shear forces and resultant damage to the endothelial cell lining. Therefore, we hypothesize that the utilization of a second-generation endovascular intervention device only may play a large role in the observed favorable outcomes in our series. Additionally, in Kellert’s research, a higher number of concurrent stenting was performed in the tirofiban group (22, 44%) versus (9, 8%) in the nontirofiban group. In contrast, we had a relatively lower rate of stenting (22%). There were also only two patients that received stents in the intracranial anterior circulation. Intracranial stenting is considered to be a high-risk procedure during treatment of symptomatic intracranial stenosis and as such, is not recommended [35]. Although the process of stent deployment may add a small degree of risk for hemorrhage, the resultant post stent cerebral hyperperfusion is more likely to occur and aggravate ischemic reperfusion injury. Thus, emergent stenting after mechanical thrombectomy could be a reason for increased risk of hemorrhage, as seen in the Kellert study. Additionally, overall lower average device passes (1.3 vs. 2), shorter device manipulation time (56 vs. 104 min) from treatment to recanalization in our study may have also contributed to favorable outcomes. Moreover, in our study, we also found the device manipulation time in ICH patients was longer than the no-ICH patient subset. Therefore, vascular injury caused by higher manipulation times during the mechanical thrombectomy process could also be an important factor leading to worse outcomes in the Kellert et al study. We speculate that the reason for previously reported higher risk of intracranial hemorrhage of tirofiban as seen in the Kellert et al study may be attributed to the operational process itself rather than effects of tirofiban administration. In the meantime, one could similarly postulate that improved operational procedure, rather than tirofiban use, may underlie the reason for improved clinical outcomes in this study compared to Kellert et al.

However, whether the operation is improved or not, the premise is that the drug itself is safe. If not, we would not have been able to get good outcomes even with good operational techniques.

In patients with anterior circulation ischemia, we found ASPECT scores in ICH patients to be lower than the no-ICH patients. The ASPECT score is a semiquantitative grading system with lower scores indicating greater infarct burden [36]. Therefore, tirofiban administration in our patients with low ASPECT scores may have contributed to be intracranial hemorrhage.

Furthermore, simultaneous to the procurement of our results, Zhao et al. in December 2017 reported the safety and effectiveness of low-dose tirofiban after MT in AIS [37]. In Zhao’s study, a low-dose tirofiban was used after vessel recanalization (0.2-0.25 mg/h) as opposed to a higher dose (6 μg/kg/h) that was used in our study. Zhao et al. hypothesized that a low dose of tirofiban may decrease the incidence of sICH and fatal ICH. However, the dosage used in Zhao’s study has not been recommended by clinical guidelines or drug instructional use within the realm of AIS for LVO. The dose of tirofiban used in our study was the routine dose indicated during angina and myocardial infarction [38]. Moreover, a single high dose of tirofiban has been shown to rapidly achieve profound degree of platelet inhibition that is maintained post procedurally as well [39] while maintaining its safety profile. As such, a low-dose of tirofiban may weaken its inherent degree of antiplatelet inhibition. Additionally, only 24% of patients were treated with rt-PA prior to administration of tirofiban in Zhao’s study vs 64.2% in our study. Zhao et al. hypothesized that overall lower rates of intravenous rt-PA may be a reason for lower incidence of sICH in the tirofiban group. However, we didn’t find any difference on rate of IV rt-PA between ICH and no-ICH patients. Administration of intravenous thrombolysis before MT is mandated as standard of care by the AHA/ASA clinical guidelines for large vessel intracranial occlusions. As such, we were able to demonstrate that the administration of the standard dose of tirofiban as a bridging therapy post MT and IV rt-PA to be safe and effective in AIS.

This prospective observational case series study is limited by lack of a comparison group undergoing mechanical thrombectomy without intravenous tirofiban. While lack of a control arm, this study did provide valuable information that serves as a benchmark to conduct further randomized control trials to analyze the safety and efficacy of intravenous antiplatelet inhibitors after mechanical thrombectomies. While we do advocate the safety and efficacy of intravenous tirofiban administration after mechanical thrombectomy in patients with AIS symptoms having received IV rt-PA, the authors would like to mention that currently the American Stroke Association guidelines do not mandate administration of antiplatelet medications within 24 hours post IV rt-PA. While ultimately it may be difficult to confirm the effectiveness of tirofiban in AIS, we were able to show that no excessive harm was associated with its use. In conclusion, intravenous administration of the standard dose of tirofiban post-Solitaire stent thrombectomy and IV rt-PA appears to be safe and relatively effective in AIS.
